# Femtosecond Laser Refractive Surgery after Descemet Stripping-Automated Endothelial Keratoplasty

**DOI:** 10.1155/2012/190953

**Published:** 2012-04-18

**Authors:** Simon Sheung Man Fung, Alfonso Iovieno, Vijay Shanmuganathan, Vicenzo Maurino

**Affiliations:** Cornea and External Diseases Service, Moorfields Eye Hospital NHS Trust, London, 162 City Road, London EC1V 2PD, UK

## Abstract

*Purpose*. To report the use of femtosecond laser-assisted in situ keratomileusis (LASIK) in the treatment of hyperopia subsequent to Descemet stripping-automated endothelial keratoplasty (DSAEK). *Methods*. Interventional case report. *Results*. A 66-year-old woman with Fuchs endothelial dystrophy developed bullous keratopathy after cataract surgery in her right eye. She underwent DSAEK with a significant postoperative hyperopic shift in her refraction. Thirteen months after DSAEK, she underwent wavefront-guided, femtosecond laser-assisted LASIK (IntraLase, Inc., Irvine, CA/AMO, Inc., IL, USA). Pretreatment unaided visual acuity was 20/120, and best-corrected visual acuity was 20/20 with a refraction of +3.25/−0.50 × 170. One year after laser refractive correction, unaided visual acuity was 20/20 with a refraction of +0.25/−0.75 × 160. *Conclusion*. To our knowledge, this is the first paper on the successful treatment of hyperopic shift related to DSAEK with wavefront-guided, femtosecond laser-assisted LASIK.

## 1. Introduction

Descemet stripping-automated endothelial keratoplasty (DSAEK) is the surgical procedure of choice for corneal endothelial diseases, allowing the surgeon to selectively replace the diseased host endothelium [1].

 The advantages of DSAEK over traditional penetrating keratoplasty (PK) include rapid visual rehabilitation, minimal-induced astigmatism maintenance of structural integrity, and predictable postoperative corneal power [2].

 The additional tissue layer in the posterior corneal surface could affect the optical characteristics postoperatively. Studies have reported significant hyperopic shift after DSAEK surgery [2]. This change is believed to be caused by the additional thickness and/or the meniscal shape of the lamellar graft inserted [3].

 Refractive surgery has been used in patients who underwent PK for correcting postoperative ametropia [4]. Femtosecond thin-flap LASIK has been shown to safely and effectively reduce postoperative refractive error, in particular the spherical component, in patients who have previously undergone PK [5].

 We hereby report the first case of treatment of hyperopia after DSAEK with wavefront-guided, femtosecond laser-assisted LASIK (IntraLASIK).

## 2. Case Presentation

A 66-year-old woman was referred to the corneal service at Moorfields Eye Hospital with intermittent blurring of vision, and a diagnosis of bilateral Fuchs endothelial dystrophy was made. She subsequently developed visually significant cataracts in her right eye and decided to undergo cataract surgery. Uncorrected visual acuity (UCVA) was 20/60 OD and 20/30 OS, with best-corrected visual acuity (BCVA) being 20/40 OD with a refraction of +1.50/+0.50 × 145 and 20/30 OS with a refraction of −0.25/+1.00 × 80. 

 Uncomplicated right phacoemulsification with implantation of a single-piece posterior chamber intraocular lens (IOL, Akreos Adapt, Bausch and Lomb UK, Ltd. Surrey, England) was performed, aiming for emmetropia. Postoperatively, examination of the right eye showed persistent bullous keratopathy, with UCVA of 20/120 and BCVA of 20/60, from day 1 to 4 months poststatus. Intraocular pressure was not raised during this period. Anterior chamber, vitreous, optic nerve, and retina were within normal limits. 

 4 months after cataract surgery, the patient underwent uncomplicated DSAEK with corneal lamellar graft prepared with a 350-micron plate Moria microkeratome (Moria, Antony, France) and a 8.25 mm trephine punch. After Descemet membrane stripping, the lamellar graft was inserted with the assistance of a Busin glide (Moria, Antony, France).

 4 months after DSAEK, the UCVA was 20/120 OD and BCVA was 20/20 with a refraction of +3.25/−0.50 × 170. Left-eye refraction remained unchanged. Slit-lamp examination showed clear cornea with DSAEK graft in good position. Patient was given a trial of soft contact lens but was intolerant and became increasingly symptomatic of anisometropia. After the alternatives for hypermetropic correction were discussed, wavefront-guided femtosecond laser-assisted LASIK was performed, 13 months after DSAEK. 

**Figure 1:**
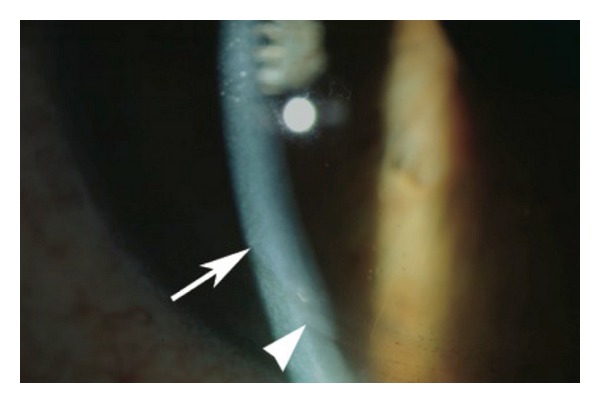
Postoperative appearance of femtosecond laser-assisted LASIK on a patient with DSAEK. The LASIK flap (arrow) and the DSAEK layer (arrowhead) are highlighted.

 The following steps were applied in the surgical procedure. The laser treatment was conducted under topical anaesthesia. Femtosecond laser (IntraLase Inc., Irvine, CA, USA) was used to create a 9.0 mm superiorlyhinged anterior lamellar flap at 110 microns. The flap was lifted and a customized wavefront (CustomVue, AMO Inc., IL, USA) ablation was performed with the S4IR (AMO Inc., IL, USA) in a 8.7 mm zone at a depth of 36 *μ*m. The flap was then replaced, and the interface was irrigated. A bandage contact lens was subsequently inserted after the flap was adhered (Figure 1). 

**Figure 2:**
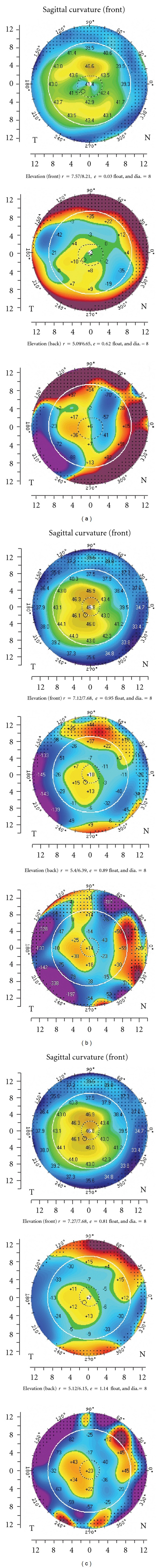
Pentacam corneal topography before (a), 7 months after (b), and 1 year after femtosecond laser-assisted LASIK (c). Note the relatively stable topography postoperatively and the substantially unchanged anterior and posterior elevation maps.

 The patient was treated with topical levofloxacin 5 mg/mL and a tapering dose of dexamethasone 0.1% over 4 weeks. A punctal plug was also inserted due to dry eye symptoms. Seven months later, the patient had UCVA of 20/20 with a refraction of +0.25/−0.75 × 160  and remained unchanged at 1 year after procedure. Endothelial cell count at the last followup was 1684 cells/mm^2^.  Figure 2 shows the topographic readings before and after refractive correction. 

## 3. Discussion

To our knowledge, this is the first paper on the application of wavefront-guided femtosecond laser-assisted LASIK for hyperopic correction in post-DSAEK patients.

 Pseudophakic bullous keratopathy is one of the commonest indications for corneal grafting. DSAEK is gaining in popularity due to its advantages over PK. However, postoperative hyperopic shift is common after DSAEK [2]. If cataract surgery was to be performed concurrently with DSAEK, the IOL power could be adjusted to compensate for the hyperopic shift [3]. 

 After DSAEK, postoperative correction with spectacles or contact lenses is often helpful but could be problematic in patients who are intolerant or those with difficulty in contact lens insertion. Other treatment modalities such as IOL exchange or sulcus piggyback IOL insertion are possible, but the risks of endothelial cell loss and corneal graft rejection with intraocular surgery render them unfavorable.

 Laser refractive surgery has been used to treat postoperative ametropia in patients who underwent PK. Both microkeratome- and femtosecond laser-assisted LASIK have been shown to be effective in the correction of post-PK ametropia, particularly the spherical component [5]. Importantly, no significant changes to endothelial cell density could be demonstrated in post-PK patients who underwent microkeratome-LASIK [6].

 Levinger et al. first reported the use of femtosecond laser-assisted arcuate keratectomy in a post-DSAEK patient for correcting postoperative astigmatism [7]. No complications were observed 7 months after procedure. A recent case series by Ratanasit and Gorovoy studied the use of microkeratome-LASIK and PRK in 5 patients with previous DSAEK, achieving good refractive outcomes without any graft rejections or the need of enhancement surgery [8].

 In our case, the patient underwent femtosecond laser-assisted LASIK 13 months after DSAEK. No complications, including graft failure, graft rejection, refractive instability, and corneal ectasia, were observed up to 1 year postoperatively. Femtosecond laser-assisted LASIK has the adventage over microkeratome LASIK of producing more predictable and uniform flap thickness, a more astigmatically neutral flap and less intraoperative epithelial injury, as shown by previous studies [9]. The effect of femtosecond laser-assisted LASIK on the corneal endothelium has also been studied in human subjects. No significant changes to endothelium cell density or hexagonality have been shown up to 1 year after laser procedure [10]. The endothelial cell count in our patient remained healthy and comparable to Ratanasit and Gorovoy's study.

 In summary, this paper suggests that femtosecond laser-assisted LASIK can be safely and effectively used in the correction of ametropia in post-DSAEK patients. Further investigations into the long-term effects of this technique are needed.
